# Students' perceptions of learning environment in an Indian medical school

**DOI:** 10.1186/1472-6920-8-20

**Published:** 2008-04-11

**Authors:** Reem Abraham, K Ramnarayan, P Vinod, Sharmila Torke

**Affiliations:** 1 Department of Physiology, Melaka Manipal Medical College (Manipal Campus), Manipal, Karnataka, India; 2 Department of Pathology, Melaka Manipal Medical College (Manipal Campus), Manipal, Karnataka, India; 3 Department of Microbiology, Melaka Manipal Medical College (Manipal Campus), Manipal, Karnataka, India

## Abstract

**Background:**

Learning environment in any medical school is found to be important in determining students' academic success. This study was undertaken to compare the perceptions of first year and clinical phase students regarding the learning environment at Melaka Manipal Medical College (MMMC) (Manipal Campus) and also to identify the gender wise differences in their perceptions.

**Methods:**

In the present study, the Dundee Ready Education Environment Measure (DREEM) inventory was used. DREEM was originally developed at Dundee and has been validated as a universal diagnostic inventory for assessing the quality of educational environment. In the present study, DREEM was administered to undergraduate medical students of first year (n = 118) and clinical phase (n = 108) and the scores were compared using a nonparametric test.

**Results:**

Among the two batches, first year students were found to be more satisfied with the learning environment at MMMC (as indicated by their higher DREEM score) compared to the clinical batch students. Gender wise, there was not much difference in the students' perceptions.

**Conclusion:**

The present study revealed that both groups of students perceived the learning environment positively. Nevertheless, the study also revealed problematic areas of learning environment in our medical school which enabled us to adopt some remedial measures.

## Background

There is an increasing interest and concern regarding the role of learning environment in undergraduate medical education in the recent years. Educational environment is one of the most important factors determining the success of an effective curriculum [1]. The quality of educational environment has been identified to be crucial for effective learning. Curriculum's most significant manifestation and conceptualization is the environment, educational and organizational which embraces everything that is happening in the medical school [2].

In the present study, Dundee Ready Education Environment Measure (DREEM) identified students' perceptions of the elements operating in the educational environment at Melaka Manipal Medical College (MMMC) (Manipal Campus). MMMC offers the Bachelor of Medicine and Bachelor of Surgery (MBBS) program which is a twinning program with Malaysia. The program runs in two phases, Phase 1 Phase 11. Students undergo Phase 1 training in Manipal, India and it comprises first year (Phase 1, Stage 1), second year (Phase 1, Stage 2) and six months of clinical training. Phase 11 component comprises clinical subjects which are taught in Malaysia. About 98% of the students are from Malaysia while the remainder are from different parts of the world.

The objectives of the present study were as follows:

1) To compare the quality of the educational environment as perceived by the first year and clinical batch students so that remedial measures could be taken to enhance students' learning experiences.

2) To identify whether there is any gender difference in the students' perceptions in first year and clinical phase.

## Methods

DREEM has been widely used as a tool to gather information about the educational environment in many institutions [3-5]. It was originally developed at Dundee and has been validated as a universal diagnostic inventory for assessing the quality of educational environment of different institutions [5].

DREEM is a 50 item inventory, consisting of 5 subscales.

a) Students' Perceptions of Learning (SPL)-12 items; maximum score is 48;

b) Students' Perceptions of Teachers (SPT)-11 items; maximum score is 44;

c) Students' Academic Self-Perceptions (SASP)-8 items; maximum score is 32;

d) Students' Perceptions of Atmosphere (SPA)-12 items; maximum score is 48;

e) Students' Social Self-Perceptions (SSSP)-7 items; maximum score is 28.

The total score for all subscales is 200.

The DREEM questionnaire was administered to students of MMMC (n = 226) in July 2006. They consisted of 118 and108 students in the first year and clinical phase respectively. The questionnaire was administered at the end of year to both the student groups on different occasions after a lecture class. In advance to administration of the questionnaire, the class was addressed regarding the purpose and process of collecting data, stressing the anonymity of the participants and the fact that the data could not be tracked to individual participants. Meanings of some of the terms such as 'course organizers' and 'registrars' were explained to the students prior to the administration of DREEM. It was also explained that the data would be used for quality assurance as well as for research purpose and their co-operation was requested. Students completed the questionnaire anonymously. As 7 students in first year and 8 students in the clinical phase did not mention their gender in the response sheet, we could only study the gender wise difference in the perceptions between 56 male and 55 female students in first year and 58 male and 42 female students in the clinical batch. Each DREEM item was scored 0 to 4 with scores of 4,3,2,1 and 0 assigned for strongly agree, agree, uncertain, disagree and strongly disagree, respectively. Reverse scoring was used for the negative items (9 items).

To pinpoint more specific strengths and weaknesses within the learning environment at MMMC, items with a mean score of 3 and above were taken as positive points and items with a mean score of 2 and below were taken as problem areas. Items with a mean score between 2 and 3 were considered as aspects of the learning environment that could be enhanced. By means of the statistical package SPSS, Mann-Whitney test was used for all the comparisons.

## Results

Table 1 shows the DREEM domain scores for the first year and clinical batch students. For Students' Perceptions of Learning, Students' Perceptions of Teachers, Students' Academic Self-Perception, Students' Perceptions of Atmosphere and Students' Social Self Perceptions, the mean domain scores for first year students were 29/48, 26/44, 19/32, 28/48 and 16/28 respectively. While for the clinical phase students, the scores were found to be 27/48, 30/44, 20/32, 30/48 and 15/28 respectively. The mean total DREEM score was found to be 119/200 for first year students and 114/200 for the clinical batch students. In general, the total DREEM domain score was found to be higher for first year students.

**Table 1:** Mean (SD) DREEM domain scores for first year and clinical batch students

**Domain**	**First year**	**Clinical batch**
Students' Perception of Learning (SPL)	29/48	27/48
Students' Perception of Teachers (SPT)	30/44	26/44
Students' Academic Self-Perception (SASP)	19/32	20/32
Students' Perceptions of Atmosphere (SPA)	28/48	30/48
Students' Social Self-Perceptions (SSSP)	16/28	15/28
Total DREEM item score for the group	119/200	114/200

Table 2 shows the mean DREEM item scores for first year and clinical batch students. It was observed that the first year students scored less than 2 for10 items (4, 5, 8, 9, 14, 25, 26, 27, 42 & 48) and above 3 for 3 items (2, 15 & 40). Clinical batch students scored less than 2 for 8 items (4, 8, 9, 14, 25, 27, 39, 42 & 48) and above 3 for one item (10). Table 3 shows the mean of items which showed statistically significant differences between the first year and clinical batch students. Out of the 13 items, 4 items (1, 2, 20, 21) were from Students' Perceptions of Learning, 4 items (2, 18, 29, 49) from Students' Perceptions of Teachers, 2 items (5, 26) from Students' Academic Self-Perceptions, 2 items (11, 33) from Students' Perceptions of Atmosphere and 1 item (15) was from Students' Social Self Perceptions.

**Table 2:** Mean (SD) DREEM item scores for first year and clinical batch students

**Domain**	**Item**	**First year**	**Clinical batch**
SPL	1. I am encouraged to participate in teaching sessions	2.77(0.79)	2.51 (0.76)
	7. The teaching is often stimulating	2.52(0.79)	2.18 (0.85)
	13. The teaching is registrar centred	2.42(1.02)	2.13 (0.99)
	16. The teaching helps to develop my confidence	2.61 (0.83)	2.60 (0.87)
	20. The teaching is well focused	2.91 (0.60)	2.58 (0.73)
	21. The teaching helps to develop my confidence	2.59 (0.79)	2.27 (0.93)
	24. The teaching time is put to good use	2.52 (0.93)	2.23(1.06)
	25. The teaching overemphasizes factual learning	1.50 (0.95)	1.57 (1.00)
	38. I am clear about the learning objectives of the course	2.87 (0.73)	2.59 (0.96)
	44. The teaching encourages me to be an active learner	2.55 (0.98)	2.31 (0.89)
	47. Long term learning is emphasized over short term learning	2.24 (1.12)	2.53 (0.96)
	48. The teaching is too teacher centred	1.85 (1.07)	1.95 (1.00)
SPT	2. The course organizers are knowledgeable	3.22 (0.68)	2.98 (0.62)
	6. The course organizers espouse a patient centered approach to consulting	1.97 (1.14)	2.16 (1.02)
	8. The course organizers ridicule their registrars	1.94 (1.03)	1.88 (0.96)
	9. The course organizers are authoritarian	1.54 (0.98)	1.57 (0.97)
	18. The course organizers appear to have effective communication skills with patients	2.22 (1.19)	2.57 (0.96)
	29. The course organizers are good at providing feedback to registrars	2.60 (0.87)	2.15 (0.95)
	32. The course organizers provide constructive criticism here	2.13 (1.03)	2.29 (0.98)
	37. The course organizers give clear examples	2.87 (0.70)	2.71 (0.84)
	39. The course organizers get angry in teaching sessions	2.05 (2.17)	1.55 (1.11)
	40. The course organizers are well prepared for their teaching sessions	3.06 (0.63)	2.89 (0.85)
	49. The registrars irritate the course organizers	2.73 (1.00)	2.20 (1.02)
SASP	5. Learning strategies which worked for me before continue to work for me now	1.86 (1.04)	2.19 (0.88)
	10. I am confident about passing this year	2.97 (2.91)	3.12 (3.16)
	22. I feel I am being well prepared for my profession	2.50 (0.85)	2.42 (1.00)
	26. Last years work has been a good preparation for this years work	1.89 (1.12)	2.54 (1.03)
	27. I am able to memorize all I need	1.39 (1.04)	1.68 (1.04)
	31. I have learned a lot about empathy in my profession	2.47 (1.05)	2.57 (0.92)
	41. My problem solving skills are being well developed here	2.46 (0.85)	2.37 (0.85)
	45. Much of what I have to learn seems relevant to a career in healthcare	2.97 (0.80)	2.73 (0.91)
SPA	11. The atmosphere is relaxed during consultation teaching	2.56 (1.03)	2.25 (0.95)
	12. The course is well time tabled	2.38 (1.11)	2.40 (1.00)
	17. Cheating is a problem in this course	2.44 (1.28)	2.38 (1.21)
	23. The atmosphere is relaxed during lectures	2.42 (1.30)	2.13 (0.98)
	30. There are opportunities for me to develop interpersonal skills	2.58 (0.88)	2.48 (0.86)
	33. I feel comfortable in teaching sessions socially	2.58 (0.75)	2.31 (0.90)
	34. The atmosphere is relaxed during seminars/tutorials	2.47 (1.04)	2.21 (1.00)
	35. I find the experience disappointing	2.37 (1.14)	2.43 (0.95)
	36. I am able to concentrate well	2.28 (0.95)	2.34 (0.92)
	42. The enjoyment outweighs the stress of studying medicine	1.76 (1.19)	1.62 (0.99)
	43. The atmosphere motivated me as a learner	2.42 (1.04)	2.41 (0.97)
	50. I feel able to ask the questions I want	2.18 (1.14)	1.96 (1.16)
SSSP	3. There is a good support system for registrars who get stressed	2.04 (1.04)	1.95 (0.78)
	4. I am too tired to enjoy this course	1.67 (1.17)	1.56 (1.13)
	14. I am rarely bored on this course	1.78 (1.00)	1.69 (1.04)
	15. I have good friends in this course	3.21 (0.78)	2.82 (1.09)
	19. My social life is good	2.53 (1.03)	2.47 (1.01)
	28. I seldom feel lonely	2.21 (1.13)	1.99 (1.22)
	46. My accommodation is pleasant	2.68 (1.00)	2.47 (1.23)

**Table 3:** Mean (SD) DREEM Inventory items where significant differences were observed between the years of study

**Items**	**First year**	**Clinical batch**	**P-value**
1. I am encouraged to participate in teaching sessions	2.77(0.79)	2.51 (0.76)	0.01
2. The course organizers are knowledgeable	3.22 (0.68)	2.98 (0.62)	0.004
5. Learning strategies which worked for me before continue to work for me now	1.86 (1.04)	2.19 (0.88)	0.017
15. I have good friends in this course	3.21 (0.78)	2.82 (1.09)	0.002
18. The course organizers appear to have effective communication skills with patients	2.22 (1.19)	2.57 (0.96)	0.009
20. The teaching is well focused	2.91 (0.60)	2.58 (0.73)	0.000
21. The teaching helps to develop my confidence	2.59 (0.79)	2.27 (0.93)	0.009
26. Last years work has been a good preparation for this years work	1.89 (1.12)	2.54 (1.03)	0.000
29. The course organizers are good at providing feedback to registrars	2.60 (0.87)	2.15 (0.95)	0.000
33. I feel comfortable in teaching sessions socially	2.58 (0.75)	2.31 (0.90)	0.01
49. The registrars irritate the course organizers	2.73 (1.00)	2.20 (1.02)	0.000

Table 4 depicts the items showing significant differences between male and female students in first year. The mean scores for female students were found to be higher for items 2 & 48 compared to the male students. In the clinical batch, none of the items showed gender wise differences. In both batches, the overall DREEM score did not show a significant gender wise difference (First year males; 118/200, females; 120/200; Clinical batch males; 114/200, females; 115/200).

**Table 4:** Mean (SD) DREEM items showing significant differences between male and female students in first year

**Items**	**Males**	**Females**	**P-value**
2. The course organizers are knowledgeable	3.12 (0.63)	3.38 (0.56)	0.01
48. The teaching is too teacher centered	1.58 (1.09)	2.09 (0.94)	0.01

## Discussion

Students were interested in completing the inventory as evidenced by the good response rate. The overall mean DREEM score for our medical school was found to be 117/200 (n = 226), indicating that, students' perceptions were more positive. The DREEM global scores for medical schools in Srilanka, Nepal, Nigeria and UK were reported as 108/200 [3], 130/200, 118/200 [6], and 139/200 [7] respectively. The mean DREEM score for a medical school in India was reported as 107.44/200 [4]. In our sample, the score for all the five domains of DREEM indicated a more positive perception both by the first year and clinical batch of students.

While taking the individual items into consideration, out of the 11 items for which the first year students scored less than 2, 5 items were negative items and belonged to the domains Students' Perceptions of Learning (25,48), Students' Perceptions of Teachers (8,9) & Students' Social Self Perceptions (4). First year students felt to a greater extent that the course over-emphasizes factual learning and is too teacher centred, compared to the clinical batch students. In the first year, students learn anatomy, physiology and biochemistry in an integrated manner. The number of independent learning sessions were less in first year compared to the clinical batch. Further, students have repeated summative assessments in first year. As the students progress to the second year and later to the clinical phase, they spent more time learning independently. Students felt that the course is stressful (item 4). The rating was more by the clinical batch students as their schedule demanded more time. Students felt that teachers were very strict and sarcastic (items 8, 9). Three items (2, 15, 40) were rated positively by the first year students. They felt that the course organizers are knowledgeable and well prepared for class. Clinical batch students felt that the teaching and learning strategies which worked for them during the preclinical phase continued to work for them and also the learning environment at MMMC seemed to make them more confident with respect to their perception regarding passing the course.

13 items (Table 3) were found to have significant difference (p < 0.01) between the two batches of students. 10 items (from all domains) (items 1, 2, 7, 11, 15, 20, 21, 29, 33, 49) were rated higher by the first year students. They felt to greater extent that teaching was stimulating enough for them to participate during teaching sessions. They also felt that teachers are knowledgeable, well focused and also prompt in providing feedback to the students. Clinical batch students felt that they were prepared well for clinical training. They also felt that the teachers communicated effectively with the patients.

Gender wise, the overall DREEM score did not show much difference in the two groups. Mayya SS reported low total DREEM score for female academic under-achievers compared to their male counterparts in a study conducted at an Indian medical school [4]. In a study reported by Hettie Till [8], the mean DREEM scores were lower for female students compared to the males.

Considering all the above observations, we arrived at the following assumptions:

Students at Melaka Manipal Medical College (Manipal Campus) felt that:

1) The course organizers are knowledgeable and well prepared for classes; but they are strict.

2) Teaching is teacher centred, and overemphasizes factual learning.

3) They are stressed.

### Remedial measures taken

1) Implementation of Problem Based Learning (PBL) sessions and Short-term Student Research Project which were intended to make the students independent learners.

2) Implementation of Personal and Professional Development (PPD) sessions and less number of summative examinations to reduce stress.

## Conclusion

The present study revealed that both groups of students perceived the learning environment positively. Nevertheless, the study also revealed problematic areas of learning environment in our medical school (as the mean score of most of the items were between 2 and 3) which enabled us to adopt some remedial measures. As the learning environment affects student motivation and achievement, it is important to get feedback from the students on how they are experiencing their learning environment.

## Competing interests

The author(s) declare that they have no competing interests.

## Authors' contributions

RA conceived the study, was involved in the data analysis, wrote the first draft of the manuscript and also contributed to the final version of the manuscript. KR was the key person in giving permission to conduct the study. He also reviewed the manuscript and contributed to the final version of the manuscript. PV and ST were involved in the data analysis and interpretation of the results. They also contributed to the final version of the manuscript.

## Pre-publication history

The pre-publication history for this paper can be accessed here:


